# Traceability of Geographical Origin in *Gentiana straminea* by UPLC-Q Exactive Mass and Multivariate Analyses

**DOI:** 10.3390/molecules24244478

**Published:** 2019-12-06

**Authors:** Zheng Pan, Feng Xiong, Yi-Long Chen, Guo-Guo Wan, Yi Zhang, Zhi-Wei Chen, Wen-Fu Cao, Guo-Ying Zhou

**Affiliations:** 1 College of Traditional Chinese Medicine, Chongqing Medical University, Chongqing 400016, China; wanguoguo1@126.com (G.-G.W.); caowenfu@hospital.cqmu.com.edu.cn (W.-F.C.); 2 Qinghai Provincial Key Laboratory of Qinghai-Tibet Plateau Biological Resources, Northwest Institute of Plateau Biology, Chinese Academy of Science, Xining 810008, China; xiongfeng1994@foxmail.com; 3 Chongqing Academy of Chinese Materia Medica, Chongqing 404000, China; zychenyl@163.com; 4 Centre for Academic Inheritance and Innovation of Ethnic Medicine, Chengdu University of Traditional Chinese Medicine, Chengdu 611130, China; letter2013@sina.com; 5 Chongqing Key Laboratory of Traditional Chinese Medicine for Prevention and Cure of Metabolic Diseases, Chongqing 400016, China; chenzw@cqmu.com.edu.cn

**Keywords:** *Gentiana straminea*, geographical origin, UPLC-Q exactive mass, metabolomics, multivariate analysis

## Abstract

The root of *Gentiana straminea* Maxim. (Gentianaceae), is officially listed as “Qin-Jiao” in the Chinese Pharmacopoeia for the treatment of rheumatic arthritis, icteric hepatitis, constipation, pain, and hypertension. To establish the geographical origin traceability in *G. straminea*, its chemical profiles were determined by a UPLC-Q exactive mass spectrometer, from which 43 compounds were identified by comparing retention times and mass spectrometry. Meanwhile, a pair of isomers (loganin and secologanol) was identified by mass spectrometry based on their fragmentation pathway. A total of 42 samples from difference habitats were determined by an UPLC-Q exactive mass spectrometer and the data were assayed with multivariate statistical analysis. Eight characteristic compounds were identified to determine the geographical origin of the herb. To estimate the key characteristic markers associated with pharmacological function, the inhibiting activities of nitric oxide (NO) production in lipopolysaccharide (LPS)-induced macrophages were examined. This finding is crucial in realizing the determination of botanical origin and evaluating the quality of *G. straminea*.

## 1. Introduction

The geographical origin and the authenticity of herbal material are often related to the safe application of their preparations, since the authenticity and quality parameters are often associated with a particular geographical origin and/or production area [1]. However, some herbal products available for purchase do not come from a fixed origin, and could fall short of quality requirements. Thus, the successful traceability of geographical origin attributes is necessary for ensuring efficacy and biosafety [2]. Metabolites are the end product of the majority of cellular processes, and, as such, are representative of the phenotype of an organism. The secondary metabolites of herbs with different geographical origins exhibit certain differences, therefore, it has been speculated that different geographical origins of medicinal herbs may be identified according to their chemical characteristics by a data mining method based on their chemical profiles [3].

The root of *Gentiana straminea* (*G. straminea*) Maxim. (Gentianaceae) is officially listed as “Qin-Jiao” in the Chinese Pharmacopoeia [4] for the treatment of rheumatic arthritis, icteric hepatitis, constipation, pain, and hypertension [5]. Phytochemical investigations have characterized the plant by the presence of a wide range of compounds, such as iridoids, secoiridoids, flavonoids, triterpenoids, alkaloids, and other types of secondary metabolites [6,7,8]. Some published methods have focused on the qualitative and quantitative determination of iridoids and secoiridoids in the plant by liquid chromatography or liquid chromatography-mass spectrometry [9,10]. Nevertheless, few of these methods have been aimed at determining the geographical origin of *G. straminea* by characteristic marker components.

In recent years, the authors of this paper successfully identified the characteristic components in different geographical origins of *Lamiophlomis rotata* by ultra-high performance liquid chromatography coupled with time-of-flight mass spectrometry (UPLC-Q/TOF/MS) [11,12,13]. Here, a comprehensive chemical composition analysis of 42 samples is proposed to evaluate the variability of *G. straminea* from different geographical origins, with sensitive, selective, and accurate UPLC-Q exactive mass spectrometer analysis. The UPLC-Q exactive mass spectrometer data was assayed to identify the characteristic components in *G. straminea* from different geographical regions. Partial least-squares discriminant analysis (PLS-DA) and orthogonal partial least-squares discriminant analysis (OPLS-DA) were employed with a metabolomic approach. To estimate the key characteristic marker associated with pharmacological function, the inhibiting activities of nitric oxide (NO) production in lipopolysaccharide (LPS)-induced macrophages were examined. In summary, the findings of this study imply that the origin of the material should be considered when it is used in traditional prescriptions and medicinal preparations.

## 2. Results

### 2.1. Identification of the Constituents in G. straminea by UPLC-Q Exactive Mass Spectrometer

**Figure 1:**
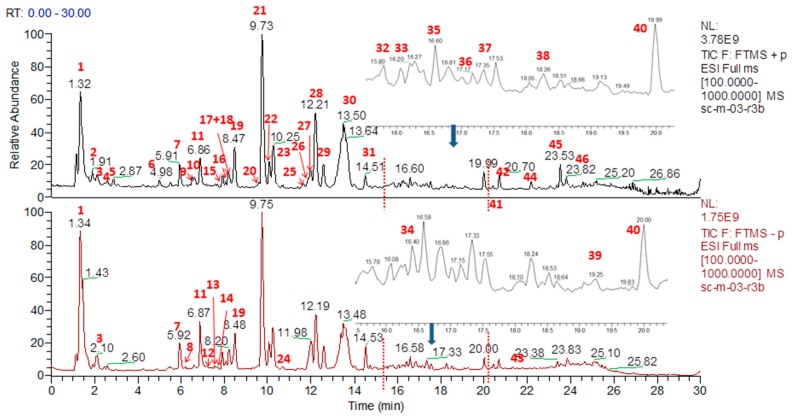
Total ion current chromatograms of substances in the extract of *G. straminea* (the no. 3 sample of Sichuan province) with positive and negative ion modes.

The no. 3 sample from Sichuan province (SC-M-03-r3b) was selected for identification by UPLC-Q mass assay as the sample showing the most peaks during 0–30 min. Forty-six peaks were detected in *G. straminea* from MS and MS^n^ in negative and positive ion mode by a UPLC-Q exactive mass spectrometer (Figure 1). The mass accuracy for all assigned components was less than 5 ppm compared with the molecular formulas of the published compounds in *G. straminea*. Gentiopicroside, swertiamarin, 6′-*O*-β-d-glucopyranosyl-gentiopicroside, sweroside, loganic acide and loganin were identified by comparison with the retention time and mass fragmentation of reference standards. The SciFinder Scholar and the PubChem databases were searched for the spectral data of other compounds reported previously in the genus *Gentiana* and *G. straminea* to identify the constituents of the herb [14,15,16,17,18,19,20]. Forty-three of these were identified by comparing the retention times and mass spectrometry, which has already been summarized [21], including 20 iridoids, 16 secoiridoids, 8 flavonoids, 2 triterpenoids, 2 lignins, 2 alkaloids, and 2 saccharides (Table 1).

Among these compounds, gentiopicroside (a type of secoiridoid) and loganin (a type of iridoid) were officially listed in the Chinese Pharmacopoeia 2015 edition for quality control of the herb [22]. In positive ion mode, secoiridoids and iridoids all showed highly abundant proton and sodium ion adducts, but a relatively lower proportion of potassium, and they also showed highly formate and chlorine ion adducts in negative ion mode. Moreover, small peaks for [2M + Na]^+^, [2M + HCOOH − H]^−^ (Supplementary Figures S1 and S2), could be observed in the spectra for positive and negative experiments. All of these mass signals were helpful in the identification of secoiridoid and iridoid components [23,24].

**Figure 2:**
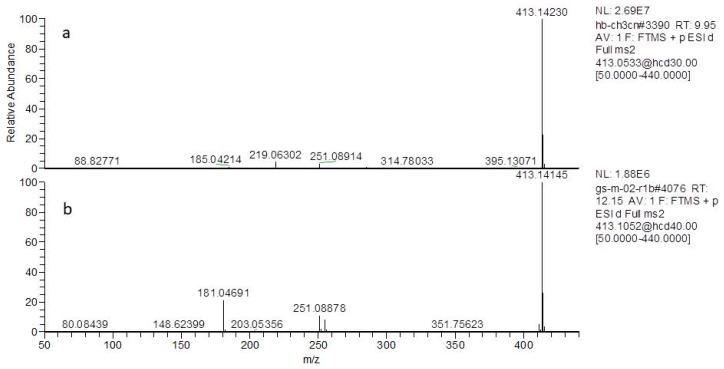
The mass and MS^2^ spectra of (**a**) loganin and (**b**) secologanol.

**Figure 3:**
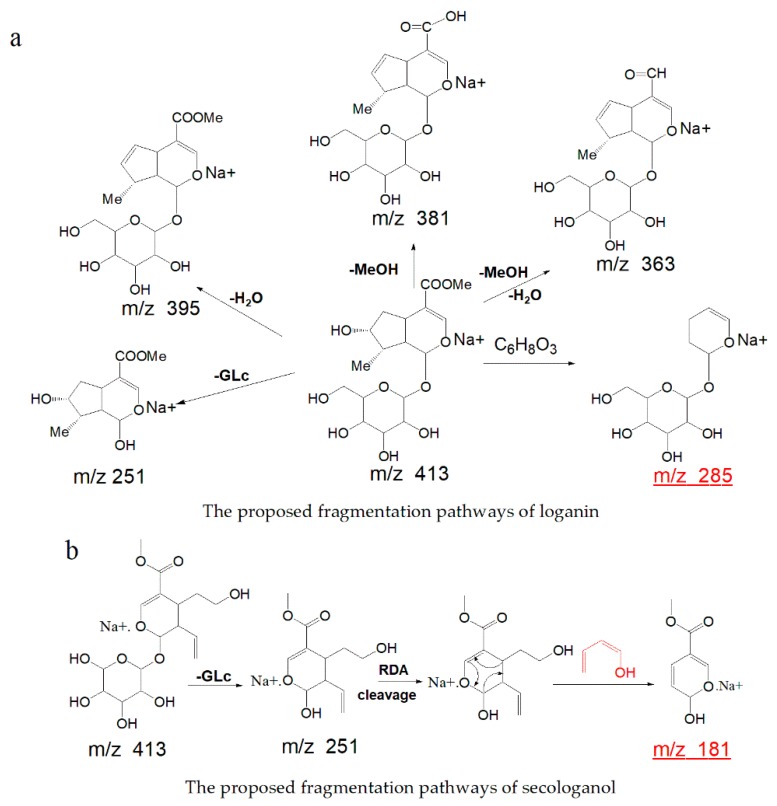
(**a**,**b**) The proposed fragmentation pathways of loganin and secologanol, respectively.

For the first time, a pair of isomers (loganin and secologanol) were identified by mass spectrometry based on their fragmentation pathway. Loganin (a type of iridoid), eluted at 9.95 min, showed fragment ions at *m*/*z* 413.14157 [M + Na]^+^ (Figure 2a) and *m*/*z* 803.29346 [2M + Na]^+^, with the elemental composition of C_17_H_26_O_10_Na (calculated 413.14240) in positive ion mode. In MS^2^, the compound formed product-characterized ions at *m*/*z* 285.09409 with the neutral loss of C_6_H_8_O_3_ (*Δm* = 128.04831 Da). It also showed ions at *m*/*z* 185.04211 Da as glucose residue adducts sodium ion, and ions at *m*/*z* 219.06264 Da indacted the compound with the neutral loss of a glucose and methanol. The proposed fragmentation pathway of the loganin is shown in Figure 3a.

Notably, secoiridoids have always produced fragmentation by Retro–Diels–Alder (RDA) cleavage of the aglycon moiety [24] and these fragmentations are quite different from that of iridoids. Secologanol (a type of secoiridoid), eluted at 12.35 min, showed fragment ions at *m*/*z* 413.141172 [M + Na]^+^ (Figure 2b) and *m*/*z* 803.29358 [2M + Na]^+^ (Supplementary Figure S1b), and after generated [M + Na − Glu]^+^ at *m*/*z* 251.08833 Da, which was identical to the aglycone fragment corresponding to the neutral loss of a glucose unit (*Δm* = 162 Da). The precursor ion produced characterized ions at *m*/*z* 181.04694 with an RDA cleavage reaction of the base skeleton and showed the neutral loss of C_4_H_6_O (*Δm* = 70 Da). The proposed fragmentation pathway of the secologanol is shown in Figure 3b.

### 2.2. Multivariate Analysis of the Global Metabolomics Data

**Figure 4:**
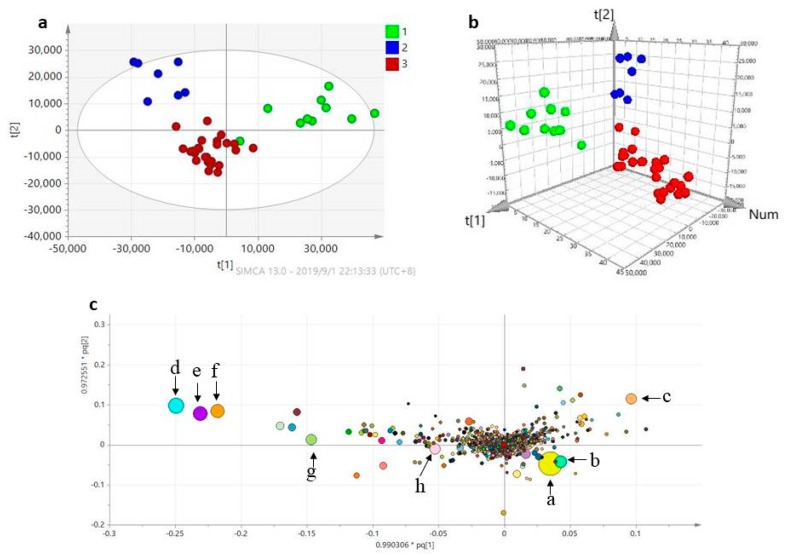
(**a**) Partial least-squares discriminant analysis (PLS-DA) score plot of *G. straminea* from three geographical origins. Green dots: samples from Gansu province, blue dots: samples from Qinghai province, red dots: samples from Sichuan province. (**b**) Orthogonal partial least-squares discriminant analysis (OPLS-DA) in 3D score plot of *G. straminea* from three rgeographical origins. Green dots: samples from Gansu province, blue dots: samples from Qinghai province, red dots: samples from Sichuan province. (**c**) Loading plot of OPLS-DA analysis of *G. straminea*. compound a: gentiopicroside (*t_R_* 9.78 min, *m*/*z* 379.09982), compound b: vitexin (*t_R_* 14.54 min, *m*/*z* 411.12610), compound c: swertiamarin (*t_R_* 8.51 min, *m*/*z* 397.11060), compound d: gentiobiose (*t_R_* 1.33 min, *m*/*z* 365.10510), compound e: sweroside (*t_R_* 10.28 min, *m*/*z* 381.11551), compound f: 2-methoxyanofinic acide (*t_R_* 22.15 min, *m*/*z* 271.08786), compound g: loganic acide (*t_R_* 6.87 min, *m*/*z* 399.12595), and compound h: 1β, α,3α,24-tetrahydroxyursa-12,20 (30)-dien-28-oic acid (*t_R_* 25.53 min, *m*/*z* 503.33640).

To globally evaluate the chemical consistency of *G. straminea* samples of different geographical origins, the UPLC-Q exactive mass datasets were subjected to partial least-squares discriminant analysis (PLS-DA) and orthogonal partial least squared discriminant analysis (OPLS-DA) to highlight differences among the *G. straminea* samples. As shown in Figure 4a, the 42 samples were roughly clustered into three groups by PLS-DA analysis. With OPLS-DA analysis, all of the samples were clearly categorized into three groups in 3D space (Figure 4b), 10 samples (green dots) from Gansu province were assigned to group I, 7 samples (blue dots) from Qinghai were assigned to group II, and 25 samples (red dots) from Sichuan province were assigned to group III.

In order to identify the most significant discriminatory features between these regions that could act as potential barcodes, an extended statistical analysis was used to provide loading score plots of OPLS-DA (Figure 4c). In this plot, according to the importance of discriminating geographical characteristics, the size and color of these points have been highlighted, as seen in Figure 4c. The eight characteristic compounds were identified as: gentiopicroside (*t_R_* 9.78 min, *m*/*z* 379.09982); vitexin (*t_R_* 14.54 min, *m*/*z* 411.12610); swertiamarin (*t_R_* 8.51 min, *m*/*z* 397.11060); gentiobiose (*t_R_* 1.33 min, *m*/*z* 365.10510); sweroside (*t_R_* 10.28 min, *m*/*z* 381.11551); 2-methoxyanofinic acid (*t_R_* 22.15 min, *m*/*z* 271.08786); 1β,2α,3α,24-tetrahydroxyursa-12,20(30)-dien-28-oic acid (*t_R_* 25.53 min, *m*/*z* 503.33640); and loganic acid (*t_R_* 6.87 min, *m*/*z* 399.12595).

In the loading score plot of OPLS-DA, it is also clearly shown that samples from Gansu province are characterized by a high content of gentiopicroside, vitexin, and loganic acid, while samples from the Sichuan habitat location had a higher relative concentration of swertiamarin, and the populations of the Qinghai province were characterized by high contents of gentiobiose, sweroside, 2-methoxyanofinic acid, and 1β,2α,3α,24-tetrahydroxyursa-12,20(30)-dien-28-oic acid.

### 2.3. Anti-Inflammatory Effect of Characterize Components

With the aid of multivariate statistical analysis, gentiopicroside was confirmed as the most characteristic marker to distinguish the geographical origin of *G. straminea*. Anti-inflammatory activity is directly associated with therapeutic effects on arthritis, thus, to further evaluate the anti-inflammatory pharmacological function of characteristic markers, the inhibiting activities of nitric oxide production were evaluated in the macrophage cell line RAW 264.7 [25].

**Figure 5:**
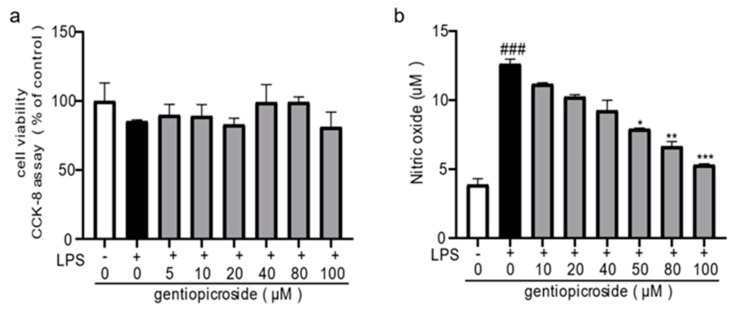
Effects of gentiopicroside on lipopolysaccharide (LPS)-induced NO production in RAW 264.7 cells. (**a**) RAW 264.7 cells were exposed to different concentrations of gentiopicroside (0, 5, 10, 20, 40, 80, and 100 μM) with or without LPS (1 μg/mL) for 24 h. Cell viability was determined by using the CCK-8 method. (**b**) RAW 264.7 cells were incubated with gentiopicroside (0, 5, 10, 20, 40, 80, and 100 μM) with stimulated by LPS (1 μg/mL) for 24 h. Extracellular levels of NO in culture media were measured using commercial Griess reagent. Data were folds of control and expressed as the mean ± SEM of six independent experiments. ^###^ *p* < 0.001 compared with the control group. * *p* < 0.05, ** *p* < 0.01, *** *p* < 0.001, compared with the LPS alone.

The cytotoxicity of gentiopicroside and LPS in RAW 264.7 cells were examined using CCK8 assay. As shown in Figure 5a, no significant difference in the viability of RAW 264.7 cells were observed among groups, suggesting that the concentrations of gentiopicroside and LPS used in the present study did not show any significant cytotoxic effects on RAW 264.7 cells. The inhibition effect of NO production induced by LPS in the macrophage-derived RAW 264.7 cells of the compound was assayed. It was found that the level of NO gradually decreased in a concentration-dependent manner in gentiopicroside. At a concentration of 100 µM, the compound significantly inhibited NO generation (83.76 ± 0.57%), with IC_50_ values of 44.2 ± 6.4 µM (Figure 5b).

In addition to gentiopicroside, nitric oxide production was also suppressed by loganic acid, swertiamarin, and vitexin. These compounds possessed the most potent inhibitory activity against NO production with IC_50_ values of 23.13 ± 5.4, 13.65 ± 7.1, and 15.71 ± 6.20 µM, respectively. All the results showed that gentiopicroside, loganic acid, swertiamarin, and vitexin were able to effectively inhibit NO production induced by LPS (1 μg/mL) in a dose-dependent manner in RAW 264.7 cells (Supplementary Figure S3). Specifically, the inhibiting effect against NO production of sweroside was also measured. However, the compound did not show inhibiting activities on LPS-induced NO production in RAW 264.7 macrophages, as has been reported [26], because the cells were incubated with sweroside for only 24 h in the experiment.

## 3. Conclusions

In the present research, a selective and specificity approach was established to illustrate the chemical composition of 42 samples in *G. straminea* with a UPLC-Q exactive mass spectrometer, and an overall chemical profile of the herb was obtained. The significant differences in metabolite compositions between three geographical origins have been identified with multivariate analyses. The anti-inflammation effects of biomarkers on LPS-induced NO production in RAW264 macrophages were examined. The results suggested that samples from Gansu province have a higher content of gentiopicroside and loganic acid, and showed better anti-inflammatory effects than others. From the legal point of view [27], the result also confirmed that samples of Gansu province have better quality than other samples. Altogether, this finding is crucial in realizing the discrimination of the botanical origin of *G. straminea*, and evaluating the herb quality.

## 4. Discussion

The objective of the current study is the development of UPLC-Q exactive mass spectrometer methodology to allow qualitative screening of geographical origin traceability in *G. straminea*. Firstly, the chemical profiles of *G. straminea* were determined with a UPLC-Q exactive mass spectrometer, from which 43 compounds were identified by comparing the retention times and mass spectrometry. Meanwhile, a pair of isomers (loganin and secologanol) was identified by mass spectrometry based on their fragmentation pathway. Although Wu, et al. had identified 30 constituents in *G. straminea* with LC-MS [9], the result was also conducive to have a comprehensive understanding of the constituents of *G. straminea*.

Secondly, 42 samples from different habitats were determined by a UPLC-Q exactive mass spectrometer and the data were assayed with multivariate statistical analysis. Gentiopicroside, vitexin, swertiamarin, gentiobiose, sweroside, 2-metho-xyanofinic acide, loganic acide, and 1β,2α,3α,24-tetrahydroxyursa-12,20(30)-dien-28-oic acid were identified as characteristic compounds to identify the geographical origin of the herb. Notably, according to the importance of these characteristic compounds, gentiopicroside was explored as the most characteristic marker to distinguish the geographical origin of *G. straminea.* Additionally, the result also confirmed the rationality of gentiopicroside as the biomarker to determine the quality of *G. straminea.* Moreover, the result indicated that samples from Gansu province would be the most suitable choice for traditional prescriptions and preparations.

It should be emphasized that, according to the Chinese Pharmacopoeia, samples from Gansu province have been shown to have higher gentiopicroside and loganic acide amounts of some compounds than others. However, samples from Sichuan province showed a higher content of swertiamarin, and pharmacological research has revealed that this characteristic compound possesses anti-diabetic and anti-hyperlipidemic effects [28], and inhibits liver fibrosis [29]. Additionally, samples from Qinghai province have shown a higher content of sweroside, which exhibited a hepatoprotective effect [30], protective effects on osteoporosis [31], and aconitine-induced cardiac toxicity effects [32]. In view of the above reasons, it remains a challenge to estimate the herb quality of different populations. Since the herb exhibits various clinical uses in traditional prescriptions, further research should be conducted to better understand its geographical origin and its associated the clinical uses.

## 5. Materials and Methods

### 5.1. Plant Materials, Reagents, and Chemicals

Forty-two wild herbs of *G. straminea* were collected around the Qinghai-Tibet plateau in Qinghai, Sichuan, and Gansu provinces during the flowering period (the locations of the samples are provided in Table 2), individuals 10 m apart from each other were sampled randomly throughout the entire range of each location. The herbs were authenticated by Professor Yi Zhang (Chengdu University of Traditional Chinese Medicine, Chengdu, China). The samples were carefully divided into roots, leaves, and inflorescences parts, and dried in the shade. The voucher samples were deposited in the College of Ethnic Medicine (Chengdu University of Traditional Chinese Medicine, Chengdu, China) and the Qinghai Key Laboratory of Qinghai-Tibet Plateau Biological Resources (Northwest Onstitute of Plateau Biology, Chinese Academy of Science, Xining, China).

Gentiopicrin (CAS:20831-76-9), loganic acid (CAS: 22255-40-9), swertamarin (CAS: 1738839-5); loganin (CAS: 18524-94-2), vitexin (CAS: 3681-93-4), sweroside (CAS: 14215-86-2), and 6′-*O*-β-d-glucopyranosylgentiopicroside (CAS: 115713-06-9) were purchased from Biopurify Phytochemicals Ltd. (Chengdu, China). The purity of all of the standards is higher than 98% (determined by HPLC), and were confirmed by the ^1^H-NMR spectra to those in the literature [14,15,16,17,18,19,20].

HPLC-grade methanol and formic acid were purchased from Merck (Darmstadt, Germany) and Tedia (Fairfield, OH, USA). Deionized water was prepared using a Millipore water treatment system (Bedford, MA, USA). Lipopolysaccharide (LPS, *Escherichia coli* 055: B5) was purchased from Sigma-Aldrich (St. Louis, MO, USA). The Cell Counting Kit-8 was purchased from Dojindo (Kyushu Japan). Griess reagents and dimethyl sulfoxide (DMSO) were purchased from Beyotime (Shanghai, China). All other reagents were of analytical grade.

### 5.2. Sample Preparation

The dried samples (100 mg of powder each) were made to the same concentration by resuspending in 5 mL of 70% aqueous methanol in an ultrasonic bath for 30 min and cooled at room temperature. The tubes were centrifuged twice at 12,000 rpm at room temperature for 5 min each time. The extraction was repeated three times using fresh aliquots of the solvent. The extracts were transferred to a 5 mL volumetric flask, which was then filled up to the final volume with extraction solvent. The sample solutions were filtered through a 0.22-μm pore size nylon membrane filter before injection into the UPLC. All samples were stored in a refrigerator at a temperature of 4 °C until analysis.

### 5.3. LC-MS/MS Analysis

The mass spectrometer Thermo Q-Exactive Plus (Thermo Scientific, San Jose, CA, USA) was equipped with heated electrospray ionization (HESI) source. Capillary temperature and vaporizer temperature were set at 330 and 280 °C, respectively, while the electrospray voltage was adjusted at 3.50 kV (operating in both positive and negative mode). Sheath and auxiliary gas were 35 and 15 arbitrary units, with an S lens RF level of 60.

The mass spectrometer was controlled by the Xcalibur 3.0 software (Thermo Fisher Scientific, San Jose, CA, USA). The exact masses of the compounds were calculated using Qualbrowser in Xcalibur 3.0 software. The mass scan range was set in the range of *m*/*z* 100–1000.

The column was a Waters Acquity UPLC BEH C18 column (100 mm × 2.1 mm, 1.7 μm particle size). The mobile phases were (a) water with 0.1% (*v*/*v*) formic acid and (b) methanol with 0.1% (*v*/*v*) formic acid. The optimized elution conditions were as follows: holding at 10% B for 2 min, a linear gradient from 10% to 13% B (all *v*/*v*) (2 to 4 min), 13% to 15% B (4 to 10 min), 15% to 17% B (10 to 15 min), 17% to 21% B (15 to 19 min), 21% to 29% B (19 to 24 min), 29% to 53% B (24 to 29 min), 53% to 75% B (29 to 35 min), 75% to 100% B (35 to 36 min), isocratic 100% B for 1 min, and then back to 7% B in 1 min. The flow rate was 0.3 mL/min. The column temperature was 35 °C. The injection volume was 2 μL.

### 5.4. Data Processing and Statistical Analysis

The data were processed according to the method described in the references [33]. The MS chromatograms spectra of 42 samples were processed for alignment, data reduction, and normalization by Xcalibur 3.0 software (Thermo Fisher Scientific, San Jose, CA, USA), the data were imported into Microsoft Excel to carry out peak area normalization after being processed by Compound Discoverer 2.0, and the processed data were exported to SIMCA-P software (ver. 13.0; Umetrics, Umeå, Sweden) for data analysis. A list of the intensities of detected peaks was generated using the retention time (*tR*) and the mass data (*m*/*z*) pairs to identify each peak. An arbitrary ID was assigned to each *tR–m*/*z* pair in the order of their UPLC elution to facilitate data alignment. This procedure was repeated for each run. Ions from different samples were considered to be identical when they had the same *tR* (tolerance within 0.01 min) and *m*/*z* (tolerance within 0.01 Da). If a peak was not detected in a particular sample, that ion intensity was recorded as zero.

### 5.5. Cell Culture and Cell Viability Measurement

Murine macrophage cell line RAW 264.7 were cultured in Dulbecco’s Modified Eagle Medium (DMEM, Hyclone Florida, USA) supplemented with 10% fetal bovine serum (FBS, ExCell Bio Shanghai, China), 1% Penicillin Streptomycin (Gibico California, USA) at 37 °C in a humidified atmosphere of 95% air and 5% CO_2_. After spreading at 80–90% confluence, cells were washed with PBS, scraped with fresh culture, and subcultured into 96-well plates at a density of 5.0 × 10^3^ cells/well and incubated with or without LPS (1 μg/mL). The cells were exposed to different concentrations of gentiopicroside, loganic acid, swertiamarin, and vitexin (0, 1, 5, 10, 20, 40, and 50 μM) with or without LPS (1 μg/mL) for 24 h. The optical density was measured at 450 nm using a multi-plate reader (BioTek, Winooski, VT, USA).

### 5.6. Nitric Oxide (NO) Assay

NO analysis was performed to evaluate inflammatory response and to measure NO release by macrophages. RAW 264.7 cells (1 × 10^5^ cells/well) were seeded in 96-well cell culture plates and allowed to adhere for 12 h. The cells were incubated on swertiamarin and loganic acid (0, 5, 10, 20, 40, 80, and 100 μM), or swertiamarin and vitexin (0, 5, 10, 20, 40, and 50 μM), respectively, with stimulation by LPS (1 μg/mL) for 24 h [34]. NO secretion by LPS-stimulated macrophages was determined by Griess reagents (Beyotime, Shanghai, China) according to the instructions of manufacturer [35]. Absorbance was measured at 540 nm and NO concentration was determined using sodium nitrite as a standard. Three replicates were carried out for each of the different treatments.

## Figures and Tables

**Table 1:** The identification of iridoid glycosides of *G. straminea* (the no. 3 sample of Sichuan province) by UPLC-Q exactive mass spectrometer.

Peak No.	RT (min)	Compound	Formula	Calculated (Da)	Selected Ion	Precursor Ion (Da)	Mass Accuracy (ppm)	Class
1	1.33	gentiobiose	C_12_H_22_O_11_	342.11622	[M + Na]^+^	365.10510	−0.25	sugars
2	1.91	gentianose	C_18_H_32_O_16_	504.16904	[M + Na]^+^	527.15784	−0.19	sugars
3	2.12	morroniside	C_17_H_26_O_11_	406.14752	[M + H]^+^	407.13815	−4.22	iridoids
4	2.41	eustomorusside	C_17_H_24_O_11_	408.12678	[M + Na]^+^	431.11603	−0.13	secoiridoids
5	2.66	miserotoxin	C_9_H_17_O_8_N	267.09542	[M + Na]^+^	290.08566	0.15	alkaloids
6	4.88	gladiatoside C1	C_29_H_26_O_12_	566.14243	[M + Na]^+^	589.13708	0.82	flavanoids
7	5.85	kingsidic acide	C_16_H_22_O_11_	390.11622	[M + Na]^+^	413.10522	−0.19	iridoids
8	6.20	secologanic acide	C_16_H_24_O_10_	374.12130	[M + Na]^+^	397.11047	−0.16	secoiridoids
9	6.49	6′-*O*-β-d-glucopyranosyl loganic acide	C_22_H_34_O_15_	538.18978	[M + Na]^+^	561.17889	−0.12	iridoids
10	6.59	6′-*O*-acetylgentiopicroside	C_16_H_24_O_12_	398.12130	[M + Na]^+^	431.15219	−0.76	iridoids
11	6.87	loganic acide	C_18_H_22_O_10_	376.13695	[M + Na]^+^	399.12595	−0.20	iridoids
12	7.10	kushenol I	C_16_H_24_O_10_	454.19916	[M + Na]^+^	477.17078	−3.81	flavanoids
13	7.29	1-*O*-β-d-gulcopyranosyl-4-epiamplexin	C_15_H_22_O_10_	362.12130	[M + Na]^+^	385.11685	1.49	secoiridoids
14	7.63	macrophylloside A	C_27_H_24_O_9_	492.14204	[M + H]^+^	493.14243	−1.50	secoiridoids
15	7.91	8-epi-kingsidic acide	C_16_H_22_O_11_	390.11622	[M + Na]^+^	413.10532	−0.17	iridoids
16	8.02	paederotoside	C_28_H_34_O_15_	633.18252	[M + Na]^+^	633.1796	0.46	iridoids
17	8.16	6′-*O*-β-d-glucopyranosyl gentiopicroside	C_22_H_30_O_14_	518.16356	[M + Na]^+^	541.15302	−0.06	secoiridoids
18	8.29	isovitexin-7-*O*-β-d-glucopyranoside	C_27_H_30_O_15_	594.15848	[M + Na]^+^	617.14746	−0.13	flavanoids
19	8.51	swertiamarin	C_16_H_20_O_10_	374.12130	[M + Na]^+^	397.11060	−0.13	secoiridoids
20	9.58	2′-acetylswertiamarin	C_18_H_24_O_11_	416.13187	[M + Na]^+^	439.12115	−0.12	secoiridoids
21	9.78	gentiopicroside	C_16_H_20_O_9_	356.11074	[M + Na]^+^	379.09982	−0.19	secoiridoids
22	9.95	loganin	C_17_H_26_O_10_	390.15260	[M + Na]^+^	413.14166	−0.18	iridoids
23	10.28	sweroside	C_16_H_22_O_9_	358.12639	[M + Na]^+^	381.11551	−0.18	secoiridoids
24	10.67	coniferin	C_16_H_22_O_8_	342.13147	[M + Na]^+^	365.11405	−1.98	lignin
25	10.85	Unknown	C_15_H_24_ON_2_	248.18886	[M + Na]^+^	271.17780	−0.32	alkaloids
26	11.85	Unknown	C_18_H_18_O_10_	394.09000	[M + Na]^+^	417.07639	−0.82	--
27	12.03	Homoorientin	C_21_H_20_O_11_	448.10057	[M + Na]^+^	471.08981	−0.12	flavanoids
28	12.35	Secologanol	C_17_H_26_O_10_	390.15260	[M + Na]^+^	413.14172	−0.16	secoiridoids
29	12.61	qinjiaoside A	C_17_H_24_O_11_	404.13187	[M + Na]^+^	427.12112	−0.13	secoiridoids
30	13.36	8-epikingisde/7-ketologanin	C_17_H_24_O_10_	388.13695	[M + Na]^+^	411.12610	−0.16	iridoids
31	14.54	vitexin/isovitexin	C_21_H_20_O_10_	432.10565	[M + Na]^+^	455.09482	−0.14	flavanoids
32	15.80	olivieroside A	C_25_H_26_O_11_	502.14752	[M + H]^+^	503.13646	−3.75	secoiridoids
33	16.20	pneumonanthoside	C_19_H_30_O_7_	370.19916	[M + Na]^+^	393.18814	−0.21	lignin
34	16.40	isoorientin-4′-*O*-glucoside	C_27_H_30_O_16_	610.15339	[M + Na]^+^	633.12128	−3.46	flavanoids
35	16.60	saprosmoside H	C_34_H_42_O_21_S	818.19394	[M + Na]^+^	841.21564	3.79	secoiridoids
36	17.17	6-p-coumaroy barlerin	C_28_H_34_O_14_	594.19486	[M + Na]^+^	617.16246	−3.60	iridoids
37	17.36	7-*O*-feruloylorientin	C_31_H_28_O_14_	624.14791	[M + Na]^+^	647.13690	−0.13	flavanoids
38	18.26	flavoconomelin	C_28_H_32_O_15_	608.17413	[M + Na]^+^	631.14191	−3.49	flavanoids
39	19.27	alboside III	C_22_H_32_O_15_	536.17413	[M + Na]^+^	559.1639	2.36	secoiridoids
40	19.99	rindoside	C_35_H_42_O_21_	798.22187	[M + Na]^+^	821.21045	−0.15	secoiridoids
41	20.40	unknown	C_20_H_30_O_5_	350.20933	[M + Na]^+^	373.19589	−0.87	--
42	20.70	triforoside	C_35_H_42_O_20_	782.22695	[M + Na]^+^	805.21564	−0.14	secoiridoids
43	21.29	oliveramine	C_20_H_20_N_2_O_4_	352.14231	[M + Na]^+^	375.12127	−2.89	alkaloids
44	22.15	2-methoxyanofinic acid	C_14_H_16_O_4_	248.10486	[M + Na]^+^	271.08786	−2.51	phenolic acides
45	23.53	1β,2α,3α,24-tetrahydroxyursa-12,20(30)-dien-28-oic acid	C_30_H_46_O_6_	502.32944	[M + H]^+^	503.33640	−0.17	triterpenoids
46	23.81	1β,2α,3α,24-tetrahydroxyurs-12-en-28-oic acid	C_30_H_48_O_6_	504.34509	[M + H]^+^	505.35211	−0.15	triterpenoids

**Table 2:** Populations of *G. straminea* from different geographical origin.

Location	Longitude (E)	Latitude (N)	Altitude (m)	No. of Samples
Gansu	101.9308–104.7733	33.36501–34.526	2905–3572	10
Qinghai	96.6487–101.7353	33.7805–33. 9361	3516–3789	7
Sichuan	98.7732–100.5236	31.0219–33.2510	3400–3796	25

## Supplementary Materials

The following are available online at https://www.mdpi.com/1420-3049/24/24/4478/s1, Figure S1: the mass spectra of loganin and secologanol in positive ion mode by UPLC-Q Exactive mass spectrometer ([2M + Na]^+^). Figure S2. the mass spectra of loganin and secologanol in negative ion mode by UPLC-Q Exactive mass spectrometer ([2M + HCOOH-H]^−^). Figure S3. Effects of loganic acid, swertiamarin, and vitexin on LPS-induced NO production in RAW 264.7 cells.
