# Supplementary material for: Traceability of Geographical Origin in Gentiana straminea by UPLC-Q Exactive Mass and Multivariate Analyses
Source: Molecules. 2019 Dec 6;24(24):4478. doi: 10.3390/molecules24244478 (PMC6943584; doi:10.3390/molecules24244478)
Supplement: Supplementary file 1 [file molecules-24-04478-s001.pdf]

Supplementary

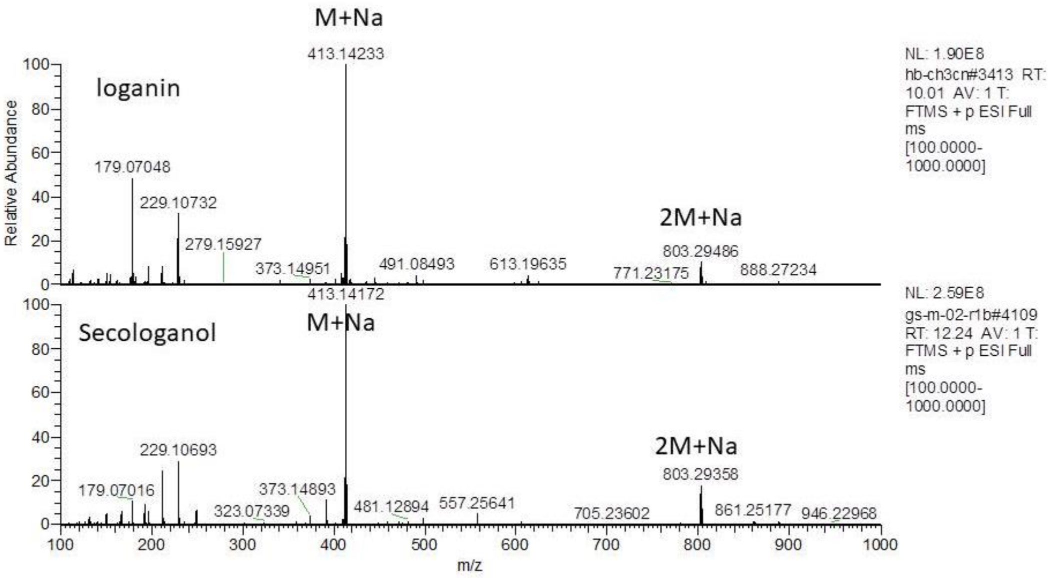

Fig.S1. the mass spectra of loganin and secologanol in positive ion mode by UPLC- Q Exactive mass spectrometer（[2M+Na] +）.

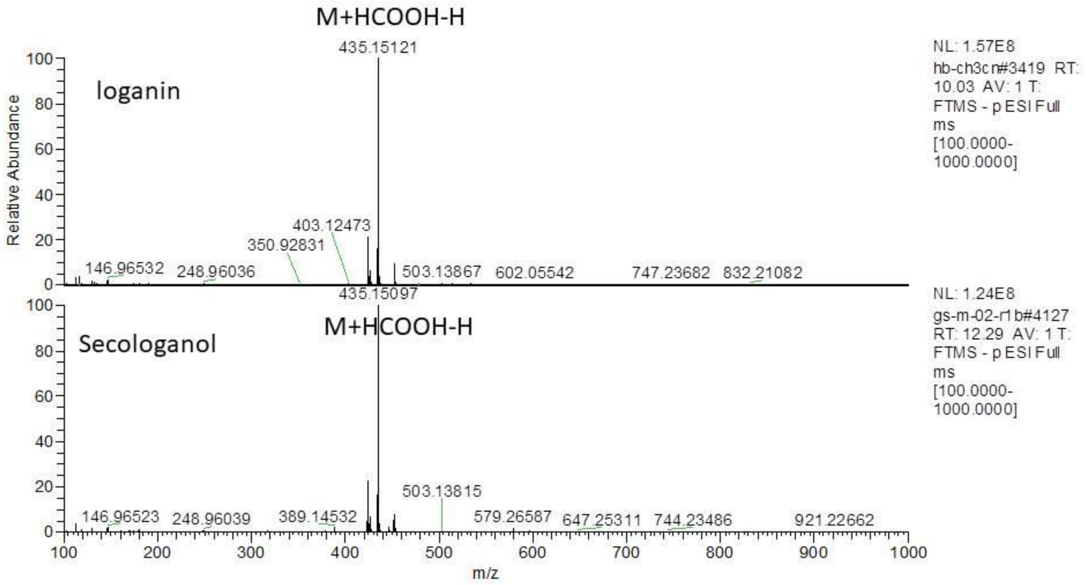

Fig.S2. the mass spectra of loganin and secologanol in negative ion mode by UPLC- Q Exactive mass spectrometer（[2M+HCOOH-H] -）.

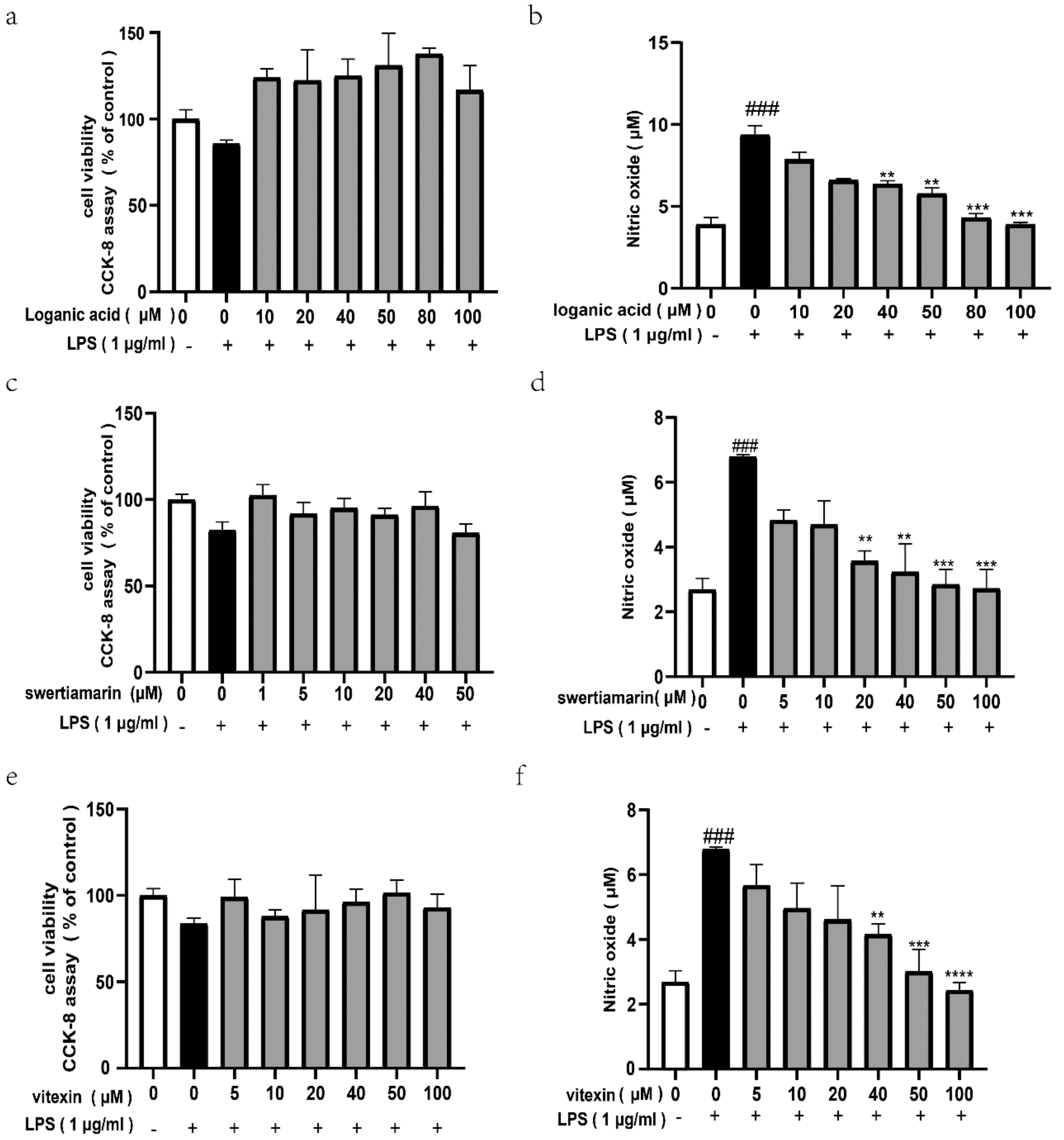

Fig.S3. Effects of loganic acid, swertiamarin, and vitexin on LPS-induced NO production in RAW 264.7 cells.

a, c and e: RAW 264.7 cells were exposed to different concentrations of loganic acid, swertiamarin and vitexin (0, 1, 5, 10, 20, 40, 50μM) with or without LPS (1 μg/ml) for 24 h. Cell viability was determined by using CCK-8 method.

b: RAW 264.7 cells were incubated with loganic acid (0, 5, 10, 20, 40, 80, 100μM) with stimulated by LPS (1 μg/ml) for 24 h. Extracellular levels of NO in culture media were measured using commercial Griess reagent. Data were folds of control and expressed as the mean ± SEM of six independent experiments.

d and f: RAW 264.7 cells were incubated with swertiamarin and vitexin (0, 5, 10, 20, 40, 50μM) with stimulated by LPS (1 μg/ml) for 24 h. Extracellular levels of NO in culture media were measured using commercial Griess reagent. Data were folds of control and expressed as the mean ± SEM of six independent experiments. ***P < 0.01, ***P < 0.001, ****P < 0.0001*, compared with the LPS alone.
